# Co-Infection of Dengue in a Pregnant Woman With COVID-19 Disease

**DOI:** 10.7759/cureus.61501

**Published:** 2024-06-01

**Authors:** Anisha Choudhary, Murari Bharadwaj, Archana Barik, Vinita Singh

**Affiliations:** 1 Obstetrics and Gynecology, Tata Main Hospital, Jamshedpur, IND; 2 Anesthesia, Tata Main Hospital, Jamshedpur, IND

**Keywords:** coronavirus, pregnancy, co-infection, dengue, covid-19

## Abstract

The pandemic due to severe respiratory syndrome coronavirus 2 (SARS-Cov-2) was one of the most damaging healthcare emergencies the world has ever seen. Co-infection with dengue virus in COVID-19-positive patients is an additional challenge especially in dengue-endemic areas. Both dengue and COVID-19 infection cause increased morbidity and adverse outcomes in pregnant women, and simultaneous infection of these two illnesses can be further detrimental and sometimes fatal in pregnant women. Here, we present a case of a pregnant woman in her early second trimester with co-infection of dengue and moderate COVID-19 disease who was managed successfully and had a favorable outcome.

## Introduction

The rapid spread of the coronavirus 2019 (COVID-19) disease resulted in one of the most damaging pandemics humans have ever encountered. In a meta-analysis of 192 studies by Allotey et al., it was concluded that women with COVID-19 disease during pregnancy are at a higher risk of developing severe diseases requiring critical care and invasive ventilation when compared to non-pregnant women of similar age group [1]. Pregnant women with COVID-19 infection are also reported to have a higher incidence of preterm birth, low birth weight, and neonatal and maternal mortality [1,2]. Dengue fever caused by the dengue virus (DENV) from the *Flaviviridae* family and transmitted via the bite of the female *Aedes aegypti* mosquito is a major public health problem in tropical and sub-tropical regions. The incidence of dengue fever has increased markedly over the last 50 years, and over half of the world’s population lives in areas at risk of dengue infection [3,4]. Studies have shown that pregnant women with dengue infection are at a higher risk of going into premature labor, have low-birth-weight babies, have higher neonatal morbidity and mortality, and have increased incidence of maternal death [5,6].

Similar presenting symptoms are shared by both dengue virus and SARS-CoV-2, and hence diagnosis may be delayed or missed leading to life-threatening complications. Pregnancy is an immunocompromised condition co-infection with COVID-19, and dengue may be fatal to both the mother and fetus [7]. Literature on co-infection of dengue in a pregnant woman with COVID-19 disease is meager, especially from eastern India. Here, we report a case of a pregnant woman with dengue and SARS-CoV-2 co-infection admitted in a tertiary care center in eastern India.

## Case presentation

**Figure 1:**
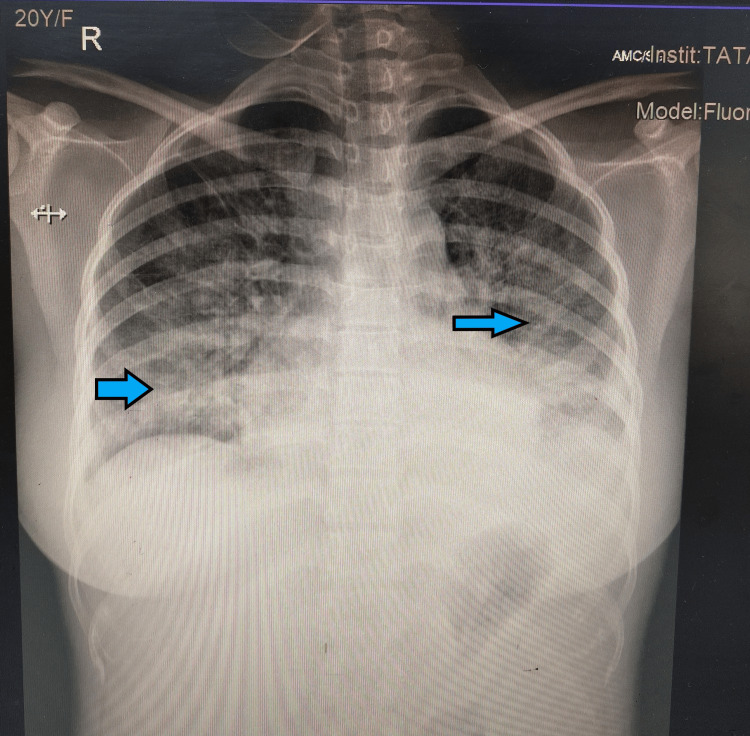
Anterior-posterior chest X-ray of the patient showing bilateral diffuse ground-glass opacities.

A 20-year-old primigravida at 16 weeks of gestation was referred from a primary care hospital with dengue nonstructural protein 1 antigen (NS1 Ag)-positive status. She complained of fever and cough for nine days and shortness of breath for one day. She also gave a history of sore throat and cough for two weeks. She was taking routine antenatal care at a district hospital, and her previous medical and surgical history was non-significant. Her vitals at the time of admission showed that the patient was febrile and had a temperature of 101°F. Her pulse rate was 118/minute, her blood pressure was 84/62 mmHg, her respiratory rate was 24 breaths/minute, and her oxygen saturation was 70%. A chest X-ray was done after admission which showed bilateral ground glass opacities, and a provisional diagnosis of COVID-19 pneumonia was made (Figure 1).

A COVID-19 rapid antigen test was done, which showed a positive result, and the patient was further managed in a COVID-19 intensive care ward. She was put on intravenous fluids, vasopressors, and paracetamol infusion. She was also started on non-invasive ventilatory support to keep her oxygen saturation at more than 94%. Her initial investigations were done which reported increased total leukocyte count, altered liver function tests, and thrombocytopenia (Table 1).

**Table 1:** Laboratory investigations of the patient on the day of admission. TLC: total leukocyte count; ALT: alanine transaminase; AST: aspartate aminotransferase; ALP: alkaline phosphatase

Parameter	On admission	Normal range
Hemoglobin (gm/dl)	9	11.5-16.5
TLC (cells per mm^3^)	16,200	4,000-11,000
Platelet (cells per mm^3^)	82,000	150,000-450,000
Prothrombin time (seconds)	11.5	11-16
International normalized ratio	1.08	0.8-1.2
Total bilirubin (mg/dl)	0.36	0.2-1
Direct bilirubin (mg/dl)	0.14	0.1-0.5
ALT (U/l)	172	5-40
AST (U/l)	86	5-45
ALP (U/l)	225	35-125
Serum creatinine (mg/dl)	0.6	0.5-1.5
Serum sodium (mEq/l)	133	136-146
Serum potassium (mEq/l)	3.8	3.5-5.5
Serum chloride (mEq/l)	98	95-108

**Figure 2:**
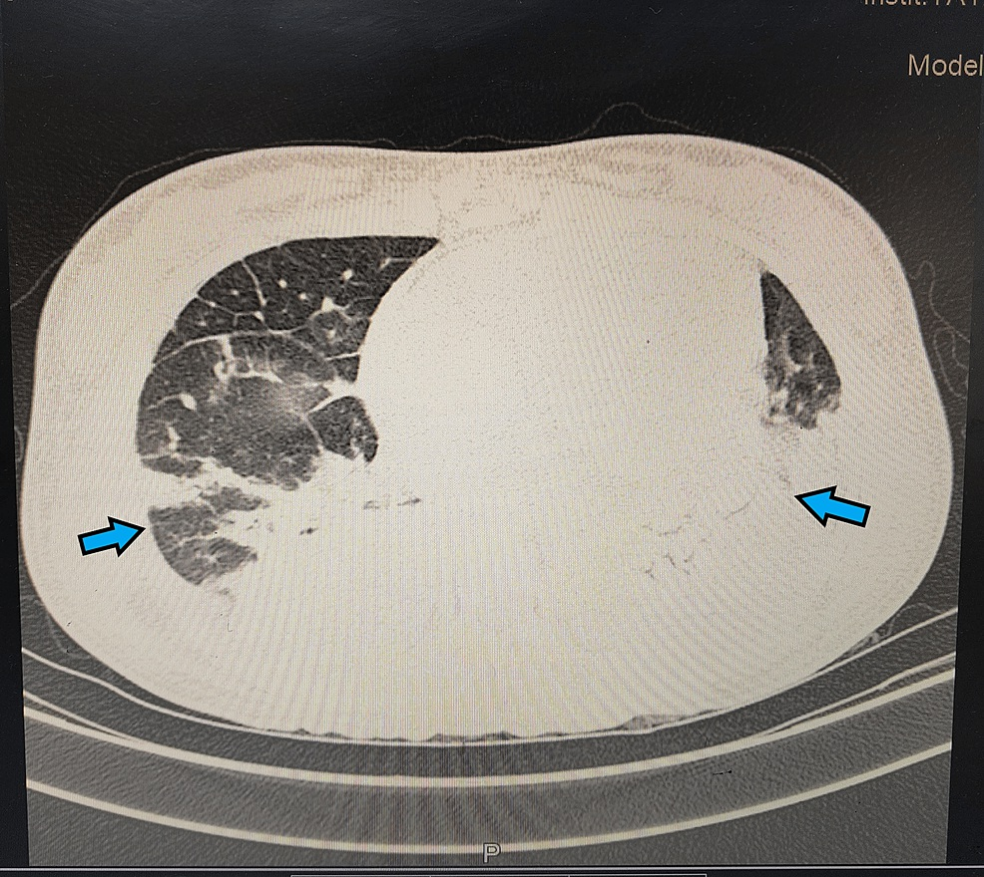
HRCT thorax showing diffuse ground glass opacities with interstitial thickening with a CT severity score of 12/25. HRCT: high-resolution computed tomography

As her total blood count was elevated, she was also started on broad-spectrum antibiotics (injection ceftriaxone 1 gm twice daily), and her urine and blood samples were sent for culture. Her high-resolution computed tomography (HRCT) thorax was done, which showed diffuse ground-glass opacities with reticular thickening, broncho-vascular thickening, and interlobular and septal thickening of bilateral lungs with regions of consolidation in the lower lobe. HRCT also showed moderate bilateral pleural effusion with minimal pericardial effusion (Figure 2). HRCT report classified the findings as COVID-19 Reporting and Data System (CO-RADS) six with a computed tomography severity score (CTSS) of 12/25.

In accordance with our hospital protocol, she was started on injection dexamethasone and injection enoxaparin for the management of COVID-19 pneumonia. An obstetric ultrasound was done, which showed a single live fetus with normal growth parameters. Her blood investigations were repeated serially (Table 2). On day three of admission, the patient was afebrile, and her platelet count was 48,000 cells per mm^3^, but as she did not have any hemorrhagic manifestations, the patient did not receive platelet transfusion. Her platelet count started to show an upward trend after the fourth day of admission. On day five of admission, she was weaned off the NIV support and maintained saturation on five liters of oxygen. Her blood culture and urine culture came back negative, and antibiotic coverage was stopped.

**Table 2:** Laboratory investigations of the patient on days of illness. HD: hospital day; TLC: total leukocyte count; ALT: alanine transaminase; AST: aspartate aminotransferase; IgM: immunoglobulin M

Parameter	HD 1	HD 2	HD3	HD5	HD7	D10	Normal Range
TLC (cells per mm^3^)	16,200	-	14,200	13,100	-	9.600	4,000-11,000
Platelet (cells per mm^3^)	82,000	65,000	48,000	58,000	92,000	1,40,000	150,000-450,000
ALT (U/l)	172	-	165	-	-	72	5-40
AST (U/l)	86	-	73	-	-	55	5-45
Serum creatinine (mg/dl)	0.6	-	-	-	-	0.58	0.5-1.5
Serum sodium (mEq/l)	133	-	135	-	-	-	136-146
Serum potassium (mEq/l)	3.8	-	3.9	-	-	-	3.5-5.5
Serum chloride (mEq/l)	98	-	106	-	-	-	95-108
Urine Culture	-	-	-	No growth	-	-	-
Dengue IgM	-	Positive	-	-	-	-	-

After six days of hospitalization, her requirement for supplemental oxygen decreased, and by the 10th day, she was completely weaned off ventilatory support. She was discharged after 10 days of hospitalization on tapering dose of tablet dexamethasone (6 mg twice a day for three days followed by 6 mg once daily for three days) and iron and calcium supplements. She did not have any residual symptoms on her routine follow-up after seven days. Thereafter, she received her antenatal care at a district hospital and delivered prematurely at 34 weeks of gestation.

## Discussion

The COVID-19 pandemic posed a huge burden on healthcare systems around the world, particularly in developing countries. The situation further deteriorates when COVID-19 coexists with other viral infections, such as the dengue fever. Dengue fever is the most prevalent mosquito-borne viral disease, and its severity can vary from mild to critical [8]. COVID-19 is a highly contagious viral illness caused by severe acute respiratory syndrome coronavirus 2 and can present as asymptomatic, mild, moderate, or severe disease [9]. Both these diseases share similar clinical symptoms, such as fever, headache, and diarrhea [10]. This overlap in clinical manifestations poses an additional challenge resulting in delayed diagnosis and management. Several case reports have highlighted serological cross-reactivity between dengue and SARS-CoV-2, which leads to false positive COVID-19 test among dengue positive patients [11]. Similarly, Lusting et al. have also reported false-positive dengue serology in 22% of COVID-19 patients [12]. Yan et al. have also described a misdiagnosed case of dengue that was later confirmed to be COVID-19 positive, resulting in fatal outcome [13].

COVID-19 and dengue fever also share biochemical parameters, such as leucopenia, thrombocytopenia, deranged liver profile, and coagulopathy, which makes identification of this condition further demanding [7]. Thrombocytopenia is one of the main manifestations of dengue virus infection and is also reported to be a clinical indicator of worsening illness and a higher risk of mortality in COVID-19 patients [14]. Agarwal et al. reported a case of COVID-19 and dengue coinfection in a pregnant woman who had refractory thrombocytopenia managed with intravenous immunoglobulins and multiple platelet transfusions [15]. Thrombocytopenia was also seen in our patient; however, it was not refractory. Serial monitoring of platelet count is important in these cases as severe thrombocytopenia may result in hemorrhage in pregnancy.

Both dengue fever and COVID-19 in pregnancy are associated with higher morbidity and mortality with an increased risk of oligohydramnios, preterm labor, and low birth weight [1,5,6]. Both dengue virus and SARS-CoV-2 can activate an inflammatory cascade and release various cytokines, such as tumor necrosis factor and interleukin-6. These immune responses may sometimes cross-react and cause cytokine storm [4]. Irwinda et al. reported a case of cytokine storm in a pregnant woman with dengue and COVID-19 co-infection, which resulted in multiorgan dysfunction and death of the patient. Adverse implications like intrauterine growth restriction, low birth weight, preterm delivery, and still birth have been reported in pregnant women with co-infection [15-17]. Our patient was diagnosed with co-infection during her early second trimester, and she had a preterm vaginal delivery.

Treatment of dengue fever and COVID-19 is mainly supportive. Thrombotic complications are common in patients with moderate to severe COVID-19 disease and are an independent predictor of poor prognosis. Therefore, anticoagulants are one of the most vital therapeutic interventions used in cases of moderate to severe COVID-19 disease [18]. Anticoagulation therapy is usually avoided in cases of dengue fever as it may increase the risk of thrombocytopenia and can even trigger Reyes syndrome [19]. Hence, anticoagulation therapy should be used in cases of coinfection after careful assessment of risk factors for thrombosis and severity of illness with regular monitoring of platelet levels. Our patient was started on injection enoxaparin as per our hospital protocol with serial monitoring of platelet levels. Intravenous steroids and immunoglobulins are also commonly used in treating COVID-19 [7], and our patient was also started on injection dexamethasone to manage her severe respiratory symptoms. However, there is no evidence of steroid use in dengue fever. Intravenous fluids, transfusion of blood products, antibiotics, and steroids are the mainstay of treatment in patients with co-infection of COVID-19 disease and dengue fever [15]. Maternal and fetal death have been reported in pregnant women with dengue and COVID-19 co-infection due to multiorgan failure and hemorrhage [7,15,17]. Although our patient suffered from severe disease and required intensive care, timely diagnosis and prompt initiation of treatment resulted in her complete recovery with a favorable maternal and neonatal outcome.

## Conclusions

Co-infection of COVID-19 and dengue in pregnancy may lead to maternal and fetal complications, such as respiratory distress, multiorgan failure, hemorrhage, miscarriage, and intrauterine fetal death. Overlapping clinical features and laboratory parameters in both infections create diagnosis and management challenges. Timely diagnosis and supportive therapy are the cornerstone of successful management of these patients. Hydration, replacement of blood components, steroids, and anticoagulant therapy have been used in different case scenarios. Further studies with a higher number of co-infected patients are required to address the prevalence of this coinfection in pregnancy and to understand its fetal and maternal implications and clinical management.
